# SOX2 is sequentially required for progenitor proliferation and lineage specification in the developing pituitary

**DOI:** 10.1242/dev.137984

**Published:** 2016-07-01

**Authors:** Sam Goldsmith, Robin Lovell-Badge, Karine Rizzoti

**Affiliations:** The Francis Crick Institute, Mill Hill Laboratory, The Ridgeway, Mill Hill, London NW7 1AA, UK

**Keywords:** SOX, Cell fate, Morphogenesis, Pituitary, Progenitor

## Abstract

*Sox2* mutations are associated with pituitary hormone deficiencies and the protein is required for pituitary progenitor proliferation, but its function has not been well characterized in this context. SOX2 is known to activate expression of *Six6*, encoding a homeodomain transcription factor, in the ventral diencephalon. Here, we find that the same relationship likely exists in the pituitary. Moreover, because *Six6* deletion is associated with a similar phenotype as described here for loss of *Sox2*, *Six6* appears to be an essential downstream target of SOX2 in the gland. We also uncover a second role for SOX2. Whereas cell differentiation is reduced in *Sox2* mutants, some endocrine cells are generated, such as POMC-positive cells in the intermediate lobe. However, loss of SOX2 here results in complete downregulation of the melanotroph pioneer factor PAX7, and subsequently a switch of identity from melanotrophs to ectopic corticotrophs. Rescuing proliferation by ablating the cell cycle negative regulator *p27* (also known as *Cdkn1b*) in *Sox2* mutants does not restore melanotroph emergence. Therefore, SOX2 has two independent roles during pituitary morphogenesis; firstly, promotion of progenitor proliferation, and subsequently, acquisition of melanotroph identity.

## Abstract (precis)

**Summary:** SOX2 has two independent roles during pituitary morphogenesis: promoting progenitor proliferation via SIX6 and determining melanotroph identity via PAX7.

## INTRODUCTION

Pituitary endocrine secretions are regulated by the hypothalamus, located within the ventral diencephalon (VD). The two organs are connected through the pituitary stalk, allowing transmission of information between the two components of the axis. The hypothalamus integrates peripheral information and regulates pituitary secretions accordingly. The gland comprises three lobes, anterior, intermediate and posterior; endocrine cell types are located in the anterior (AL) and intermediate (IL) lobes, whereas the posterior lobe comprises hypothalamic axon termini and glial cells. Secreted hormones control the function of other endocrine glands, and also different tissues. They are required for normal function of the organism as they intervene in many different physiological processes, including growth, reproduction, metabolism and response to stress. Consequently, deficits are associated with significant morbidity (Kelberman et al., 2009). It is therefore important to understand the role of genes and their proteins whose mutations are associated with pituitary hormone deficiencies, in order to characterize the aetiology of such deficiencies, improve diagnosis and, in consequence, treatments.

Mutations in the gene encoding the HMG-box transcription factor SOX2 are associated with pituitary hormone deficiencies, or hypopituitarism, both in humans and mice (Kelberman et al., 2008, 2006). SOX2 is present from the earliest stages of pituitary development, in cells of the pituitary anlagen or Rathke pouch (RP) and is maintained in the post-natal and adult gland (Fauquier et al., 2008). Its expression defines a population of pituitary progenitor/stem cells (SC) throughout all these stages (Andoniadou et al., 2013; Rizzoti et al., 2013). However, the function of SOX2 within such cells is poorly defined.

We, and others, have shown that SOX2 is involved at several levels for hypothalamo-pituitary axis development and function. It is required in the VD for specification of the future hypothalamus and for the infundibulum, which is essential for both induction and maintenance of RP and will later give rise to the pituitary stalk and posterior lobe (Zhao et al., 2012; Trowe et al., 2013). SOX2 is also required for the development of IL lobe tumors in mice mutant for P27 (also known as CDKN1B) (Li et al., 2012). In the embryo, the IL is specified in the dorsal part of RP. PAX7, which is expressed from 15.5 days post-coitum (dpc), acts as a pioneer transcription factor for emergence of melanotrophs, the sole endocrine cell type populating this lobe (Budry et al., 2012). These secrete melanocyte-stimulating hormone (MSH) to regulate pigmentation. MSH is proteolytically cleaved from pro-opiomelanocortin (POMC), which also gives rise to adrenocorticotropic hormone (ACTH), secreted by AL corticotrophs. In the embryonic gland, P27, which is mostly known as a cell cycle negative regulator but also performs several other functions (Godin and Nguyen, 2014), is required to prevent cell cycle re-entry in differentiated cells (Bilodeau et al., 2009), whereas post-natal pituitary tumors develop in *p27*-null mice, exclusively within the IL (Fero et al., 1996; Nakayama et al., 1996). P27 has been shown to recruit co-repressors to downregulate *Sox2* expression (Li et al., 2012). We previously demonstrated the relevance of this genetic interaction and the role of SOX2 in tumor development, by showing that deletion of one allele of *Sox2* in *p27^−/−^* mice prevented occurrence of IL tumors (Li et al., 2012).

In this report, we characterize the role of SOX2 during pituitary morphogenesis. Because of its important role in the VD, conditional loss-of-function approaches are necessary to study specific functions of SOX2 during pituitary development. Here, we have used four *Cre* drivers to conditionally delete the gene in RP, while maintaining its expression in the VD, allowing phenotypic analysis of early and late phenotypes. We first demonstrate that SOX2 is required for normal levels of cell proliferation in RP. This is in agreement with Jayakody et al. (2012), but we go on to reveal that *Sox2* deletion results in a complete downregulation of SIX6, known for its role in RP progenitor proliferation (Li et al., 2002). We then demonstrate a second role for SOX2. Deletion of the gene results in a reduction in endocrine cell differentiation, but we still observe some hormone-secreting cells. In particular, some POMC-positive cells are present in the developing IL of *Sox2* mutants. However, we show here that these are not melanotrophs, but ectopic corticotrophs, and that this can be explained by a complete downregulation of the melanotroph cell fate factor PAX7 in the absence of *Sox2*. We further demonstrate that this phenotype is independent of the rate of cell division. These results therefore demonstrate a sequential requirement for SOX2 during pituitary morphogenesis, firstly to promote progenitor proliferation, and secondly for IL cell fate acquisition. This might also underlie its role in IL tumor development.

## RESULTS

### Deletion of *Sox2* in RP results in reduction of progenitor proliferation

SOX2 is expressed throughout RP at 10.5 dpc, becoming gradually restricted to the cells lining the cleft as development progresses (Fauquier et al., 2008). To understand the role of the protein during pituitary development, we deleted the gene using two different *Cre* drivers, *Foxg1^Cre^* (Hebert and McConnell, 2000) and *Nkx3.1^Cre^* (Y.P.H., S. M. Price, Z. Chen, W. A. Banach-Petrosky, C. Abate-Shen and M. M. Shen., unpublished).

**Fig. 1.**
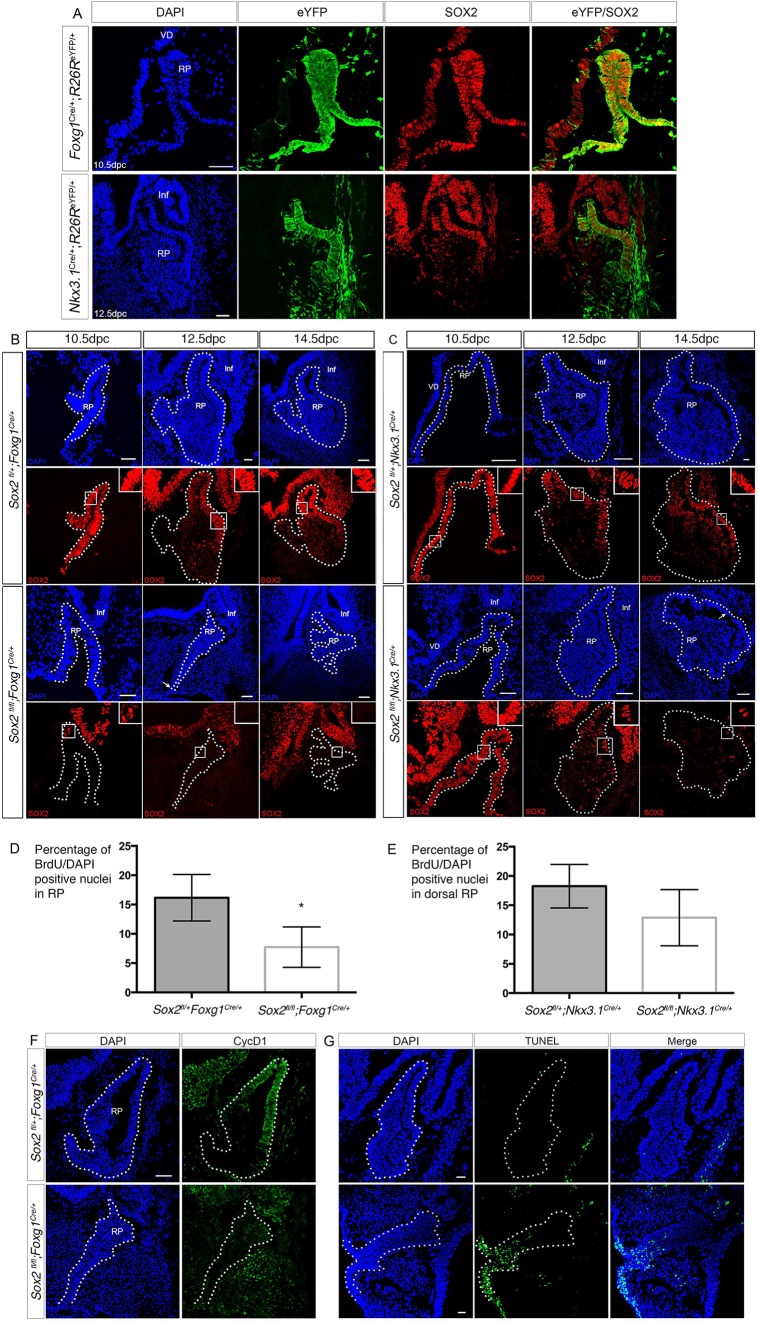
**Loss of SOX2 results in a reduction of RP progenitor proliferation.** (A) *Foxg1^Cre^* and *Nkx3.1^Cre^* lineage-tracing analysis. Immunofluorescence for eYFP and SOX2. In *Foxg1^Cre/+^;R26R^eYFP/+^* embryos at 10.5 dpc, the reporter displays a ubiquitous activity in RP. At 12.5 dpc in *Nkx3.1^Cre/+^;R26R^eYFP/+^* embryos, eYFP is observed in the future IL. 69.6±6.5% (mean±s.d.) of SOX2-positive cells express eYFP (*n*=3) in *Nkx3.1^Cre/+^;R26R^eYFP/+^* embryos at this stage. (B,C) Immunofluorescence for SOX2 on mutant embryos. SOX2 downregulation after *Cre* recombination is initially detectable at 10.5 dpc in *Sox2^fl/fl^;Foxg1^Cre/+^* embryos (B) and 12.5 dpc in *Sox2^fl/fl^;Nkx3.1^Cre/+^* embryos (C). Deletion of *Sox2* using *Foxg1^Cre^* results in formation of a hypomorphic pouch at 12.5 dpc, still attached to the oral ectoderm (arrow, B). Later deletion with *Nkx3.1^Cre^* is initially associated with a thinner dorsal pouch at 14.5 dpc (arrow, C). (D,E) Analysis of cell proliferation after *Sox2* deletion in RP. After a 1 h pulse at 12.5 dpc, the percentage of BrdU^+^; DAPI^+^ nuclei is lower in *Sox2^fl/fl^* compared with *Sox2^fl/+^* embryos. BrdU incorporation is significantly reduced when *Foxg1^Cre^* is used to delete *Sox2* (*Sox2^fl/+^;Foxg1^Cre/+^*: 16.2±2%, *n*=4 compared with *Sox2^fl/fl^;Foxg1^Cre/+^*:7.7±2.0%, *n*=3; *P=0.03). Using *Nkx3.1^Cre^*, proliferation is less affected (*Sox2^fl/+^;Nkx3.1^Cre^*^/+^: 18.3±1.9%, *n*=4 and *Sox2^fl/fl^;Nkx3.1^Cre/+^*: 12.9±2.4%, *n*=4; ns). (F) Immunofluorescence for cyclin D1 at 12.5 dpc. Cyclin D1 delineates the dorsal proliferative region in *Sox2^fl/+^;Foxg1^Cre/+^* RP; its expression is significantly reduced in homozygous *Sox2^fl/fl^;Foxg1^Cre/+^* embryos. (G) TUNEL assay at 12.5 dpc. There is persistence of a patch of apoptotic cells in the hypoplastic *Sox2^fl/fl^;Foxg1^Cre/+^* RP where it is still abnormally connected to the oral ectoderm. All sections are sagittal. Dotted outline indicates RP. Scale bar: 50 μm in A-C,G; 100 μm in F. RP, Rathke's pouch; VD, ventral diencephalon; Inf, infundibulum.

*Foxg1* is ubiquitously expressed in RP (Xuan et al., 1995). Accordingly, a lineage-tracing experiment using the *R26R^eYFP^* allele revealed eYFP expression throughout RP in *Foxg1^Cre/+^;R26R^eYFP/+^* embryos at 10.5 dpc (Fig. 1A). By 18.5 dpc, all cells in the pituitary appear eYFP positive (Fig. S1A).

*Nkx3.1* is expressed in RP from 10.5 dpc until at least 14.5 dpc, but is restricted to the dorsal region (Treier et al., 1998). In *Nkx3.1^Cre/+^;R26R^eYFP/+^* embryos, eYFP is first detected in a few cells in RP at 10.5 dpc (Fig. S1B), becoming substantially upregulated in the dorsal RP at 12.5 dpc, where SOX2 is also predominantly present (Fig. 1A). By the end of gestation, reporter activity is mostly observed in the IL, but there is also a significant contribution of eYFP-positive cells in AL (Fig. S1B).

When we used either *Foxg1^Cre^* or *Nkx3.1^Cre^* to delete *Sox2*, downregulation of the protein closely matches the activity profile of the *Cre* drivers (Fig. 1B,C). Expression of SOX2 is almost completely extinguished in RP at 10.5 dpc in *Sox2^fl/fl^;Foxg1^Cre/+^* embryos. Notably, morphological abnormalities, including hypoplasia and failure to separate from the underlying oral ectoderm, are observed at 12.5 dpc (Fig. 1B). In *Sox2^fl/fl^;Nkx3.1^Cre/+^* embryos, SOX2 expression is downregulated later, at 12.5 dpc (Fig. 1C). Pituitary hypoplasia is observed as a consequence at 14.5 dpc, mostly in the dorsal region of RP (Fig. 1C; Fig. S3A). Most *Sox2^fl/fl^;Nkx3.1^Cre/+^* and all *Sox2^fl/fl^;Foxg1^Cre/+^* animals die shortly after birth. This could be consecutive to hypopituitarism, and notably to a deficiency in ACTH, which would be expected in the in *Sox2^fl/fl^;Nkx3.1^Cre/+^* animals because the pituitary is one essential organ where this driver is active and *Sox2* expressed (Schneider et al., 2000); we did not, however, examine other tissues where the driver is active and SOX2 is essential. When using *Foxg1^Cre^*, post-natal lethality could be due to hypopituitarism, but there are also severe forebrain defects (Ferri et al., 2013).

The RP hypoplasia, observed when both *Nkx3.1^Cre^* and *Foxg1^Cre^* are used to delete *Sox2*, could be caused by a defect in proliferation, an increase in apoptosis, or both. Progenitor proliferation was examined by performing a one-hour BrdU incorporation pulse at 12.5 dpc. A reduction in the percentage of dorsal proliferating progenitors was observed in homozygous mutant *Sox2^fl/fl^;Nkx3.1^Cre/+^* RPs, compared with *Sox2^fl/+^;Nkx3.1^Cre/+^* embryos but this did not reach statistical significance (Fig. 1E). When *Sox2* was deleted using *Foxg1^Cre^*, a further significant reduction in BrdU incorporation was observed throughout RP in embryos homozygous for the conditional allele versus those that were heterozygous (Fig. 1D). We then examined expression of the cell cycle promoter cyclin D1 and observed a clear downregulation of the protein in *Sox2^fl/fl^;Foxg1^Cre/+^* embryos (Fig. 1F) suggesting that the cell cycle was stopped at the G_1_/S checkpoint.

**Fig. 2.**
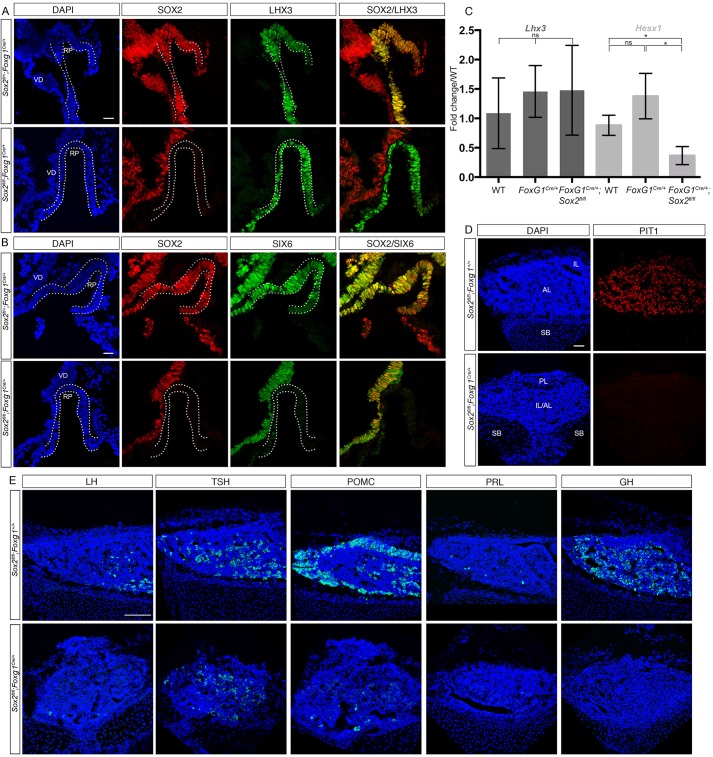
**Loss of SOX2 is associated with downregulation of the transcription factors SIX6 and HESX1, and a later dramatic reduction in cell numbers of all hormonal lineages.** (A) Immunofluorescence for SOX2 and LHX3 at 10.5 dpc. SOX2 and LHX3 are co-localized in all cells of RP in *Sox2^fl/+^;Foxg1^Cre/+^* RP. In *Sox2^fl/fl^;Foxg1^Cre/+^* embryos, LHX3 expression appears unaffected by the loss of SOX2. (B) Immunofluorescence for SOX2 and SIX6 at 10.5 dpc. SOX2 and SIX6 are co-localized in all cells of RP in controls. In *Sox2^fl/fl^;Foxg1^Cre/+^* embryos, SIX6 expression is dramatically downregulated in RP. Dotted outline indicates RP. (C) Quantification of *Lhx3* and *Hesx1* mRNA expression levels by real-time PCR. Embryonic heads (hindbrain was excluded as *Lhx3* is expressed there; Zhadanov et al., 1995) were analyzed at 11.5 dpc. There is a significant downregulation of *Hesx1* expression levels between wild-type and *Sox2^fl/fl^;Foxg1^Cre/+^* mutant embryos (*P=0.0178, *n*=3 in each group) and between *Foxg1*^*Cre/+*^ and *Sox2*^*fl/fl*^*;Foxg1*^*Cre/+*^ mutant embryos (*P=0.0135, *n*=3 in each group), whereas, in agreement with the immunofluorescence results in A, *Lhx3* is not significantly affected (ns). Results presented as mean±s.d. (D) Immunofluorescence for PIT1 at 15.5 dpc. PIT1 is expressed throughout the AL in *Sox2^fl/+^;Foxg1^+/+^* embryos. In mutant *Sox2^fl/fl^;Foxg1^Cre/+^* embryos, PIT1 expression is essentially lost. (E) Immunofluorescence for pituitary hormones at 16.5 dpc. There is a dramatic reduction in all differentiated endocrine cell types in *Sox2^fl/fl^;Foxg1^Cre/+^* embryos, with no discernable separation between anterior and intermediate lobes. The prolactin antibody used here has been reported to display a low cross-reactivity to POMC; as prolactin is only detectable after birth, we therefore believe that the staining observed in the control might represent corticotrophs. Scale bar: 25 μm in A,B; 50 μm in C; 100 μm in D; 200 μm in E. RP, Rathke's pouch; VD, ventral diencephalon; IL, intermediate lobe; AL, anterior lobe; SB, sphenoid bone.

We examined apoptosis by performing TUNEL assays at 12.5 dpc. In *Sox2^fl/fl^;Nkx3.1^Cre/+^* embryos, we did not observe any significant difference with controls (data not shown). In contrast, in *Sox2^fl/fl^;Foxg1^Cre/+^* embryos there is a ventral zone in the area of the hypoplastic RP that is still attached to the oral ectoderm where cells are undergoing apoptosis (Fig. 1G). At 11.5 dpc, in wild-type embryos, apoptosis occurs in the same region and this is thought to result in separation of RP from the underlying oral ectoderm (Charles et al., 2005). In *Sox2^fl/fl^;Foxg1^Cre/+^* embryos at 12.5 dpc, we observe more apoptosis in this ventral domain. However, the hypoplastic pouch does not appear to separate from the ectoderm, even at a later stage (Fig. 2D). This suggests that ventral apoptosis is not the only process required for separation of RP and that hypoplasia perturbs this event.

In conclusion, *Sox2* deletion in RP is associated with reduced proliferation and downregulation of cyclin D1 expression, in agreement with a previous study (Jayakody et al., 2012). Earlier and more efficient deletion of the gene, using *Foxg1^Cre^* instead of *Nkx3.1^Cre^*, results in a more severe phenotype, with formation of a hypoplastic RP that fails to separate from the underlying ectoderm.

### Ubiquitous *Sox2* deletion in RP results in the complete loss of SIX6 expression, downregulation of *Hesx1* transcripts, and a deficiency in most pituitary endocrine cell types

To better characterize the pathways and factors involved downstream of SOX2 in RP progenitor proliferation, we performed a marker analysis in *Sox2^fl/fl^;Foxg1^Cre/+^* mutants. We first examined expression of LHX3, a LIM homeodomain transcription factor necessary for progenitor maintenance (Sheng et al., 1996). Its expression in *Sox2^fl/fl^;Foxg1^Cre/+^* RP appears unaffected at 10.5 dpc, and is similar to that observed in *Sox2^fl/+^;Foxg1^Cre/+^* embryos (Fig. 2A). Transcripts for PITX2, another homeodomain transcription factor required for maintenance of RP progenitors (Charles et al., 2005; Gage et al., 1999), also appear unaffected by the loss of SOX2 in *Sox2^fl/fl^;Foxg1^Cre/+^* embryos at 12.5 dpc (Fig. S2A).

Pituitary hypoplasia and reduced proliferation are phenotypic features observed in embryos null for the homeobox gene *Six6* (Li et al., 2002). These two features, with varying degrees of severity, are also seen in *Sox2^fl/fl^;Nkx3.1^Cre/+^* and *Sox2^fl/fl^;Foxg1^Cre/+^* mutants (Fig. 1B,C). At 10.5 dpc, SOX2 and SIX6 are co-localized throughout RP in *Sox2^fl/+^;Foxg1^Cre/+^* embryos (Fig. 2B). However, in mutant *Sox2^fl/fl^;Foxg1^Cre/+^* embryos SIX6 expression is dramatically downregulated at 10.5 dpc, coincident with loss of SOX2 (Fig. 2B).

HESX1 is a paired homeodomain transcription factor functioning as a co-repressor and required for forebrain and pituitary development (Andoniadou et al., 2007; Dasen et al., 2001). It has been proposed that SOX2 could directly participate in *Hesx1* regulation because it can bind the *Hesx1* promoter and induce its transcriptional activation *in vitro* (Kelberman et al., 2006). We therefore quantified *Hesx1* expression using real-time PCR with RNA extracted from whole heads at 11.5 dpc, as the gene is exclusively present in RP at this stage, before any significant morphological defect appears in *Sox2^fl/fl^;Foxg1^Cre/+^* embryos. In *Sox2^fl/fl^;Foxg1^Cre/+^* embryos we observe a significant reduction of more than 50% of *Hesx1* transcript levels compared with wild-type embryos (Fig. 2C). To assess the specificity of this result we examined *Lhx3* transcripts levels in the same samples. We observed no significant difference in *Lhx3* expression levels, in agreement with our previous results showing that expression of its protein product is not affected (Fig. 2A).

We also examined the expression of the paired-like homeobox transcription factor PROP1, which is upregulated as HESX1 expression fades in RP and is necessary for emergence of the PIT1 lineage and gonadotrophs (Gage et al., 1996; Sornson et al., 1996). Expression of *Prop1* is clearly downregulated in mutant embryos at 12.5 dpc (Fig. S2A) as previously observed (Jayakody et al., 2012). At 15.5 dpc, as endocrine cell differentiation takes place, we observe a dramatic reduction of PIT1 (also known as POU1F1) protein expression (Fig. 2D), and a loss of most hormonal cell types at 16.5 dpc (Fig. 2E), whereas *Sox2^fl/fl^;Nkx3.1^Cre/+^* embryos show a less dramatic but clear reduction in differentiated cell types (Fig. S2B). In contrast, we found proportionally more thyrotrophs in *Sox2^fl/fl^;Foxg1^Cre/+^* than any other endocrine cell types. As PIT1 is completely downregulated, we hypothesize that these represent PIT1-independent, transient thyrotrophs emerging at 12.5 dpc, before PIT1-dependent definitive thyrotrophs arise at 15.5 dpc (Kelberman et al., 2009). These are normally localized rostrally; however, in *Sox2^fl/fl^;Foxg1^Cre/+^* embryos the morphology of RP is severely disrupted, to the extent that any delineation between the IL and AL lobes is no longer discernable. As thyrotrophs are the primary endocrine cell type remaining in RP, loss of tissue organization results in these cells becoming spread both rostrally and caudally throughout the gland. Moreover, we observe in a proportion of mutants a disruption of the basisphenoid cartilage, probably because of the maintenance of an abnormal connection with the oral ectoderm (Fig. 1G). This leads to ectopic location of pituitary tissue in continuity with the oral ectoderm (Fig. 2D) (Jayakody et al., 2012).

In summary, downregulation of *Six6*, *Hesx1* and *Prop1* in mutants suggests that SOX2 participates in regulation of the expression of these three factors, which are normally co-expressed with SOX2 and are necessary for correct development of RP (Dasen et al., 2001; Gage et al., 1996; Li et al., 2002; Sornson et al., 1996; Yoshida et al., 2009). Consequently, very little endocrine cell differentiation takes place in *Sox2^fl/fl^;Foxg1^Cre/+^* hypoplastic pituitaries, where essentially only some early differentiating thyrotrophs are observed. The three genes could be direct or indirect targets of SOX2, but we hypothesize that *Six6* regulation is likely to be direct, as we previously showed this to be the case in the ventral diencephalon (Lee et al., 2012).

### SOX2 is expressed at low levels in IL melanotrophs and is not required for SIX6 expression in these cells

**Fig. 3.**
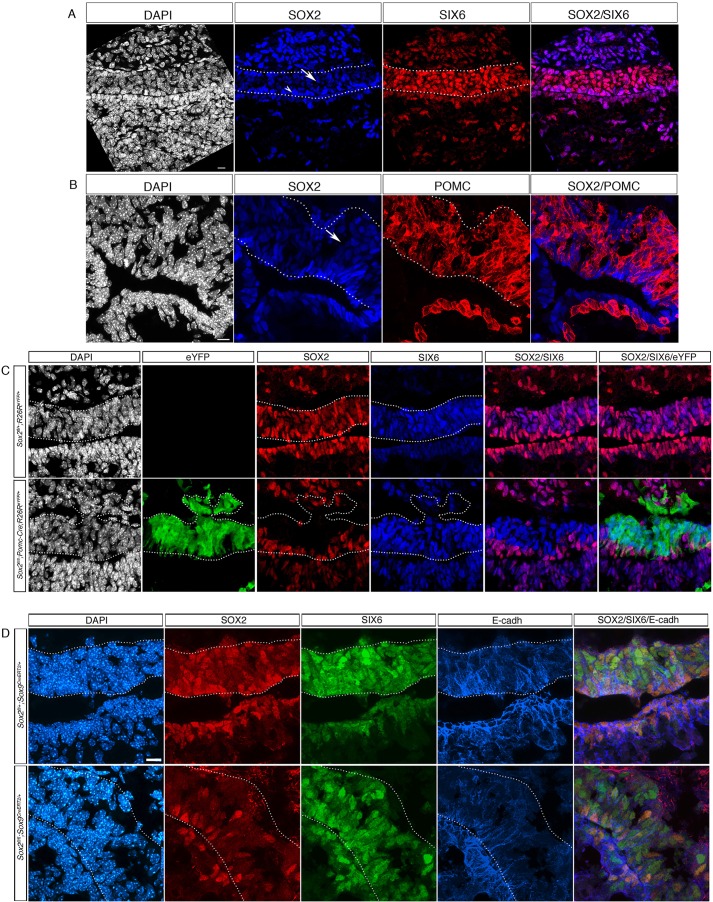
**SOX2 regulates SIX6 expression exclusively in the SOX2^Hi^ progenitor population.** (A) Immunofluorescence for SOX2 and SIX6 at 18.5 dpc in a control embryo. SOX2 is highly expressed in cells lining the pituitary lumen (SOX2^Hi^; arrowhead), and at lower levels in cells within IL (SOX2^Low^; arrow). Both SOX2-positive cell populations uniformly express SIX6. (B) Immunofluorescence for SOX2 and POMC at 18.5 dpc in a control embryo. SOX2^Low^ cells are POMC-positive melanotrophs (arrow). (C) Immunofluorescence for SOX2, SIX6 and eYFP in a *Pomc-Cre;Sox2^fl/fl^;R26R^eYFP/+^* embryo at 18.5 dpc. SOX2^Low^ expression is specifically lost in eYFP-positive melanotrophs, whereas eYFP-negative, SOX2^Hi^ cells are maintained around the cleft. SIX6 expression in IL is unaffected by the loss of SOX2*.* (D) Immunofluorescence for SOX2, SIX6 and E-cadherin in *Sox2^fl/+^;Sox9^CreErt2/+^* and *Sox2^fl/fl^;Sox9^CreErt2/+^* embryos at 18.5 dpc, induced by tamoxifen at 13.5 dpc. SOX2 expression is lost in some E-cadherin-positive cells in the epithelial cell layer lining the cleft. Deletion of SOX2 does not result in downregulation of SIX6. Scale bar: 10 μm in A,B,D; 5 μm in C. IL is outlined.

By the end of gestation, at 18.5 dpc, SOX2-positive cells are mainly found surrounding the lumen of the IL and AL, although some are also scattered in the AL parenchyma (Fig. 3A,B) (Fauquier et al., 2008). These cells, highly positive for SOX2 (SOX2^Hi^), do not express any hormones and at least a proportion of them represent SCs (Andoniadou et al., 2013; Rizzoti et al., 2013). In the IL, SOX2 is maintained throughout the lobe, albeit at lower levels than in the epithelial stem/progenitor cell layer lining the cleft (Fig. 3A). Cells expressing low levels of SOX2 in the IL (SOX2^Low^) are POMC-positive melanotrophs (Fig. 3B). These are the only differentiated cells in the pituitary to maintain nuclear expression of SOX2. This appears to break with the usual assumption that SOX2 is associated with stem cells, even though there are many exceptions (Ekonomou et al., 2005; Hoefflin and Carter, 2014). It is also a finding that has received little attention. We therefore decided to focus our analysis on the role of SOX2 in IL.

At 18.5 dpc, SIX6 and SOX2 remain co-expressed but, in contrast with SOX2, the levels of SIX6 expression appear uniform in both the cells lining the cleft and in melanotrophs (Fig. 3A). We set out to investigate whether, as we observed in RP, SOX2 is required for SIX6 expression at this later stage.

We first examined whether SOX2 is necessary for SIX6 expression within terminally differentiated melanotrophs (SOX2^Low^; SIX6^+^; POMC^+^ cells). We used *Pomc-Cre*, which is expressed in all melanotrophs and in a proportion of corticotrophs (Langlais et al., 2013), to delete *Sox2* (Fig. 3C). In *Sox2^fl/fl^;Pomc-Cre; R26R^eYFP/+^* embryos at 18.5 dpc, SOX2 expression is efficiently lost in eYFP-positive cells in the body of the IL, whereas expression is maintained in the eYFP^–^; SOX2^Hi^ cells that line the lumen. SIX6 expression appears unaffected by the loss of SOX2 in IL eYFP-positive cells (Fig. 3C).

We further investigated the SOX2-SIX6 interaction by deleting SOX2 in undifferentiated cells, using *Sox9^CreErT2^*, which, like *Sox9* itself, is expressed from 14.5 dpc in pituitary stem/progenitor cells (Rizzoti et al., 2013). At 18.5 dpc, E-cadherin expression is enriched in progenitors lining the pituitary cleft (Chauvet et al., 2009). We therefore assessed deletion of SOX2 by examining expression of the protein in E-cadherin-positive cells. We did not observe any obvious difference in E-cadherin expression between *Sox2^fl/+^;Sox9^CreErT2^* and *Sox2^fl/fl^;Sox9^CreErT2^* at 18.5 dpc (Fig. 3D). However, following CreER2 induction, SOX2 is absent in a proportion of E-cadherin-positive cells lining the cleft in *Sox2^fl/fl^;Sox9^CreErT2^* pituitaries, whereas SIX6 expression appears unaffected by the loss of SOX2 (Fig. 3D). This could simply mean that SIX6 expression does not rely on SOX2 at this stage, in progenitors/SCs. Nevertheless, the identity of SCs might be altered as *Sox2* is deleted. Therefore SIX6^+^; SOX2^–^ cells might not be progenitors and/or SCs anymore but differentiated and/or differentiating cells in which maintenance of SIX6 is independent of SOX2, as we show in IL POMC-positive cells (Fig. 3C).

### Loss of SOX2 in IL results in downregulation of PAX7 and a switch in IL cell fate from melanotrophs to corticotrophs

**Fig. 4.**
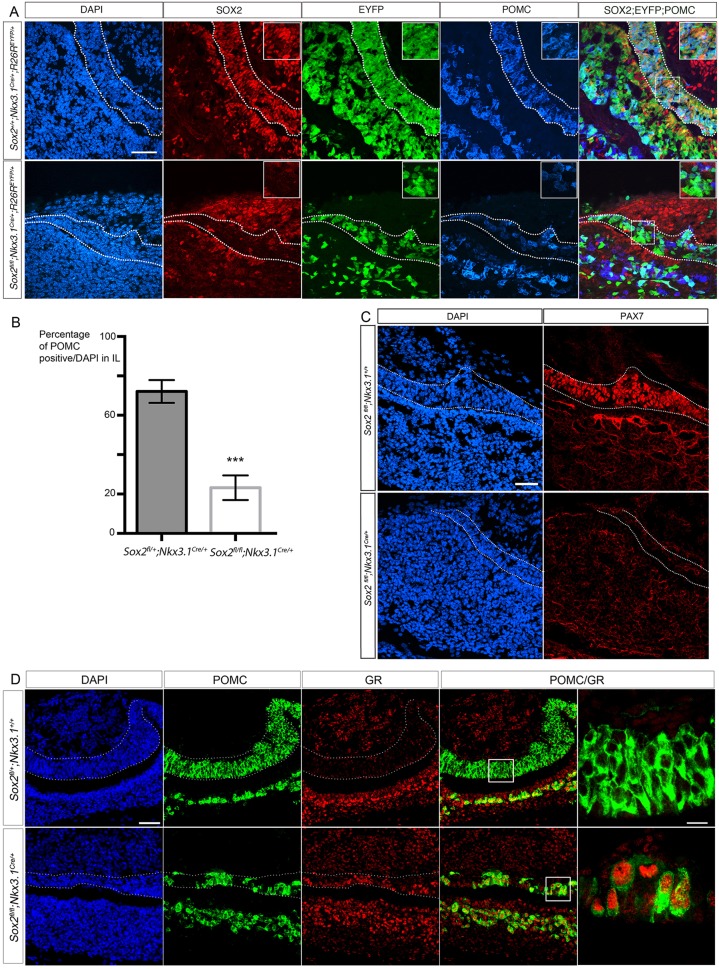
***Sox2* deletion in IL progenitors results in reduction of POMC-positive cells, complete downregulation of PAX7, and ectopic differentiation of corticotrophs.** (A) Immunofluorescence for SOX2, eYFP and POMC at 18.5 dpc in *Nkx3.1^Cre^* mutants. POMC is present in SOX2^Low^ cells in the IL of *Sox2^+/+^;Nkx3.1^Cre/+^;R26R^eYFP/+^* embryos. In mutant *Sox2^fl/fl^;Nkx3.1^Cre/+^;R26R^eYFP/+^* embryos, SOX2 is essentially lost and the number of POMC-positive cells reduced. (B) Percentage of POMC-positive cells in the IL of *Sox2^fl/fl^;Nkx3.1^Cre/+^* embryos at 18.5 dpc (23.2±6.2% of DAPI-positive cells, *n*=3), is significantly lower (***P=0.0008) than in heterozygous *Sox2^fl/+^;Nkx3.1^Cre/+^* embryos (72.1±5.8%, *n*=3). Results presented as mean±s.d. (C) Immunofluorescence for PAX7 at 18.5 dpc. PAX7 is expressed exclusively in the IL in *Sox2^fl/+^;Nkx3.1^+/+^* pituitaries. Expression is dramatically downregulated in *Sox2^fl/fl^;Nkx3.1^Cre/+^* IL. (D) Immunofluorescence for POMC and glucocorticoid receptor (GR), at 18.5 dpc in *Nkx3.1^Cre^* mutants. GR is normally present in POMC-positive corticotrophs in AL, but not in POMC-positive IL melanotrophs, as observed in *Sox2^fl/+^;Nkx3.1^Cre/+^* pituitaries. In mutant *Sox2^fl/fl^;Nkx3.1^Cre/+^* pituitaries, GR is ectopically present in the IL POMC-positive cells, demonstrating that these are in fact corticotrophs. Scale bars: 50 μm in A,C,D; 5 μm for magnifications in D. IL is outlined.

To better characterize the IL phenotype in *Sox2* mutants, we focused our analysis on *Sox2^fl/fl^;Nkx3.1^Cre/+^* embryos where this lobe is still discernable, in contrast with *Sox2^fl/fl^;Foxg1^Cre/+^* embryos where the phenotype is more severe. Deletion of *Sox2* in the dorsal region of RP at 12.5 dpc in *Sox2^fl/fl^;Nkx3.1^Cre/+^* embryos results in reduced proliferation, a severe reduction in the size of the dorsal RP, and subsequently IL at 18.5 dpc (Fig. 1C,E, Fig 4A; Fig. S2). This is associated with a significant reduction in the percentage of POMC-positive cells in IL (Fig. 4B).

To investigate the deficit in POMC-positive cells in the IL, we examined the expression of factors required for the emergence of melanotrophs. We first examined the expression of TPIT (also known as TBX19), which is required for POMC activation in both melanotrophs and corticotrophs (Pulichino et al., 2003). In the IL of *Sox2^fl/fl^;Nkx3.1^Cre/+^* embryos, its expression is dramatically downregulated, mirroring the loss of POMC (Fig. S3). In *Tpit*-null pituitaries, IL cells can change fate and become ectopic PIT1-independent thyrotrophs or SF1-positive gonadotrophs (Pulichino et al., 2003). We did not observe any ectopic expression of TSH or SF1 in the *Sox2^fl/fl^;Nkx3.1^Cre/+^* IL, demonstrating that IL cells did not adopt an alternative thyrotroph or gonadotroph fate in mutants (Figs S2, S3). The paired homeodomain protein PAX7 is a pioneer transcription factor required for melanotroph fate (Budry et al., 2012). It is expressed exclusively in the IL, becoming upregulated just prior to TPIT at 15.5 dpc. In *Sox2^fl/fl^;Nkx3.1^Cre/+^* embryos at 18.5 dpc we observe a complete loss of PAX7 expression (Fig. 4C). This result suggests that the *Sox2*-deleted, POMC-negative cells in the mutant IL fail to commit to the melanotroph fate. In addition, the complete loss of PAX7 raises questions about the identity of the POMC-positive cells present in the mutant IL.

A switch in IL cell fate from melanotrophs to corticotrophs has been observed in *Pax7*-null pituitaries (Budry et al., 2012). This phenotype is characterized by ectopic expression of the glucocorticoid receptor (GR) in POMC-positive, *Pax7*-null IL cells. GR is normally excluded from melanotrophs and confined to glucocorticoid-responding cells of the AP, including POMC-positive corticotrophs (Budry et al., 2012). We therefore examined expression of GR and observed that it is ectopically expressed in the IL of *Sox2^fl/fl^;Nkx3.1^Cre/+^* embryos, co-localizing with POMC (Fig. 4D). This strongly suggests that the SOX2-deleted, POMC-positive IL cells are in fact ectopic corticotrophs that have switched fate in the absence of SOX2, and consequently PAX7. This implies a specific requirement for SOX2 in IL cell fate.

### SOX2 is not required for maintenance of PAX7 expression

**Fig. 5.**
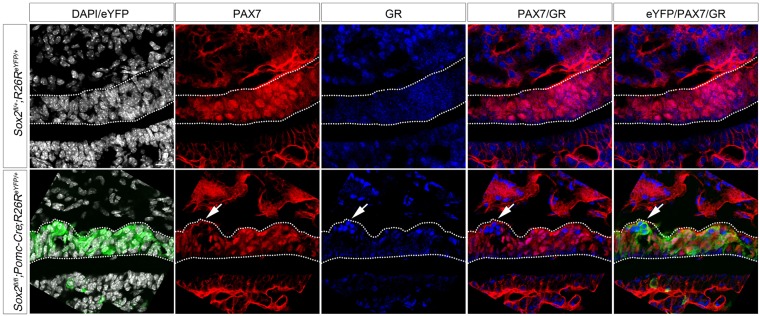
***Sox2* deletion in IL after cell commitment does not affect melanotroph cell fate.** Immunofluorescence for PAX7, eYFP and GR, at 18.5 dpc in *Pomc-Cre* mutants. PAX7 is expressed in nearly all cells of the IL in both *Sox2^fl/+^* and *Sox2^fl/fl^;Pomc-Cre;R26R^eYFP/+^* embryos. GR is not present in IL in *Sox2^fl/fl^;*Pomc-Cre;*R26R^eYFP/+^* embryos. There is, however, a patch of cells that are negative for PAX7 (arrow) and these ectopically express GR in mutant *Sox2^fl/fl^;Pomc-Cre;R26R^eYFP/+^* embryos. These likely represent cells where ectopic deletion of *Sox2* with the *Pomc-Cre* transgene occurred before endogenous *Pomc* expression (Fig. S4). IL is outlined.

To better characterize the interaction between SOX2 and PAX7, we examined *Pomc-Cre;Sox2^fl/fl^;R26R^eYFP/+^* embryos, where *Sox2* is deleted exclusively in differentiated melanotrophs and corticotrophs (Langlais et al., 2013) (Fig. 3C). We did not observe a loss of PAX7, or ectopic GR expression in these mutants (Fig. 5), suggesting that SOX2 is required for initiation rather than maintenance of PAX7 expression.

However, we still detect a small number of ectopic corticotrophs, negative for PAX7 and positive for GR (Fig. 5, arrow)*.* We therefore analyzed in detail the activity of the *Pomc-Cre* transgene by examining *Pomc-Cre;R26R^eYFP/+^* embryos. At 14.5 dpc, which is before the initiation of endogenous POMC expression at 16.5 dpc, we observe a few eYFP-positive cells in the *Pomc-Cre;R26R^eYFP/+^* IL, demonstrating early ectopic expression of the *Pomc-Cre* transgene (Fig. S4). *Sox2* is therefore deleted ectopically in these cells before PAX7 is expressed at 15.5 dpc, resulting in the differentiation of ectopic corticotrophs, as observed in *Sox2^fl/fl^;Nkx3.1^Cre/+^* mutants. Taken together, these data further reinforce the suggestion that SOX2 is required for initiation and not maintenance of PAX7 expression.

### SOX2 regulates progenitor proliferation and melanotroph cell lineage specification independently

The adoption by a proportion of cells of an alternative corticotroph cell fate in the absence of SOX2 in IL, where the protein is normally maintained in differentiated cells, led us to hypothesize that SOX2 has two independent functions. Firstly, SOX2 is required for the generation of a sufficient number of progenitors (Fig. 1), and secondly, for the specification of melanotroph fate by inducing PAX7 expression (Fig. 4). To verify this hypothesis, we decided to restore proliferation in *Sox2^fl/fl^;Nkx3.1^Cre/+^* mutants and examine melanotroph differentiation.

We observed, as reported previously (Jayakody et al., 2012), a slight upregulation of the cell cycle negative regulator P27 in *Sox2* null mutants (data not shown). In the developing pituitary P27 has been demonstrated to prevent re-entry of differentiated cells into the cell cycle. In its absence, cell differentiation is not perturbed but there is ectopic proliferation (Bilodeau et al., 2009). We therefore examined whether proliferation and melanotroph cell differentiation were restored in *p27^−/−^;Sox2^fl/fl^;Nkx3.1^Cre/+^* embryos.

**Fig. 6.**
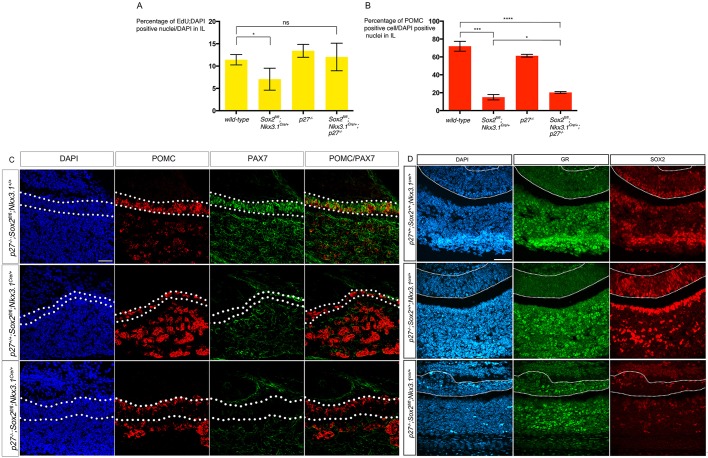
**Deletion of *p27* in *Sox2^fl/fl^;Nkx3.1^Cre/+^* embryos result in a rescue of IL cell proliferation, but melanotroph cell fate acquisition is not restored.** (A) Analysis of cell proliferation in IL. The percentage of EdU^+^;DAPI^+^ nuclei in IL was quantified at 18.5 dpc after a 1 h pulse. There is a significant (*P=0.026) decrease in the percentage of cells that incorporate EdU between wild-type (11.4±1.2%, *n*=4) and *Sox2^fl/fl^Nkx3.1^Cre/+^* embryos (7.1±2.4%, *n*=3). In contrast, in double mutants, percentages of EdU-positive nuclei (12.03±3.1%, *n*=3) are not significantly different from wild-type (ns, *P*=0.76). Therefore, ablation of *p27* restores normal levels of proliferation in mutants. (B) Analysis of cell fate acquisition in IL. 72±5.5% (*n*=3) of cells in the IL are POMC-positive in wild-type embryos. Both *Sox2^fl/fl^Nkx3.1^Cre/+^* and *p27^−/−^;Sox2^fl/fl^Nkx3.1^Cre/+^* embryos display significantly lower percentages (15±3%, *n*=3, ***P=0.0001 and 20.3±1%, *n*=4, ****P<0.0001) but there is a significant difference between single and double mutants (*P=0.019) demonstrating a slight rescue of the differentiation defect. Results presented as mean±s.d. (C) Immunofluorescence for POMC and PAX7 at 18.5 dpc. PAX7 and POMC appear normally expressed in the IL of *p27^−/−^;Sox2^fl/fl^;Nkx3.1^+/+^* embryos. In contrast, expression of both proteins is downregulated in *Sox2^fl/fl^;Nkx3.1^Cre/^*^+^ embryos, as previously observed, and also in *p27^−/−^;Sox2^fl./fl^Nkx3.1^Cre/+^* embryos. (D) Immunofluorescence for GR and SOX2 at 18.5 dpc. GR is not present in the IL of *p27^−/−^;Sox2^+/+^;Nkx3.1^Cre/+^* but it is still ectopically expressed in *p27^−/−^;Sox2^fl/fl^;Nkx3.1^Cre/+^* embryos. There is a noticeable increase in SOX2 staining in *p27^−/−^;Sox2^+/+^;Nkx3.1^Cre/+^*, in agreement with previous data demonstrating upregulation of *Sox2* in absence of p27 (Li et al., 2012). Scale bars: 50 μm. IL is outlined.

We first explored proliferation levels in *p27^−/−^;Sox2^fl/fl^;Nkx3.1^Cre/+^* pituitaries following a 1 h pulse of EdU at 18.5 dpc (Fig. 6A). We observed a significant reduction in EdU incorporation in *Sox2^fl/fl^;Nkx3.1^Cre/+^* pituitaries compared with wild-type. In contrast, there was no significant reduction in EdU incorporation in *p27^−/−^;Sox2^fl/fl^;Nkx3.1^Cre/+^* embryos compared with wild-type, demonstrating a rescue of proliferation in double mutants (Fig. 6A). In agreement with these results, we observe a thicker IL in *p27^−/−^;Sox2^fl/fl^;Nkx3.1^Cre/+^* compared with *Sox2^fl/fl^;Nkx3.1^Cre/+^* embryos (Fig. 6C).

We then quantified the percentage of POMC-positive cells in IL (Fig. 6B). We found a significant reduction in the percentage of POMC-positive cells in the IL of both *Sox2^fl/fl^;Nkx3.1^Cre/+^* and *p27^−/−^;Sox2^fl/fl^;Nkx3.1^Cre/+^* ILs compared with wild-type embryos. However there is a slight, but significant, increase in the percentage of POMC-positive cells in the IL of *p27^−/−^;Sox2^fl/fl^;Nkx3.1^Cre/+^* embryos compared with *Sox2^fl/fl^;Nkx3.1^Cre/+^* embryos, demonstrating a partial rescue of the differentiation defect.

We then examined PAX7 and POMC expression, and found both to be unaffected in *p27*-null embryos, with no re-activation of PAX7 in *p27^−/−^;Sox2^fl/fl^;Nkx3.1^Cre/+^* pituitaries (Fig. 6C). As a consequence, ectopic expression of GR is observed, which demonstrates the presence of ectopic corticotrophs, (Fig. 6D).

Therefore, despite restoring proliferation and improving the proportion of differentiated cells, the melanotroph lineage specification defect is still present. These results further argue for an independent role of SOX2 in RP progenitor proliferation and subsequently in acquisition of IL cell fate.

## DISCUSSION

Congenital defects in pituitary development and function can be associated with substantial morbidity and/or deficiencies that compromise quality of life. These can pose significant challenges for treatment, especially when linked to other clinical problems. For example, heterozygous loss-of-function mutations in *SOX2* in humans are associated with severe eye defects, ranging from micropthalmia to anopthalmia, but affected individuals also exhibit other congenital defects including hypopituitarism that can affect everything from height to puberty (Macchiaroli et al., 2014; Suzuki et al., 2014). However, the role of SOX2 in the developing pituitary is not well understood. Here, we have explored this in the mouse, revealing the role of the protein in cell proliferation and differentiation and defining several crucial downstream genes.

To characterize the role of SOX2 in the developing pituitary we have conditionally deleted the gene using two different *Cre* drivers. Deletion using *Foxg1^Cre^*, expressed early and ubiquitously in RP (Wang et al., 2010), showed that SOX2 is required for normal levels of proliferation in RP progenitors. This is in agreement with Jayakody et al. (2012) who deleted *Sox2* using *Hesx1^Cre^*, which displays a pattern of activity very similar to that of *Foxg1^Cre^*. The generation of a reduced pool of progenitors severely impairs pituitary development and emergence of endocrine cell types is consequently dramatically reduced. This is supported by the slight but significant improvement in IL cell differentiation observed after rescue of cell proliferation by removing the cell cycle inhibitor P27 (see below). *Cre* driven by *Nkx3.1* is active later than when it is driven by *Foxg1*, moreover, it is mostly restricted to the dorsal part of RP, the future IL. RP development is less perturbed in *Sox2^fl/fl^;Nkx3.1^Cre/+^* compared with *Sox2^fl/fl^;Foxg1^Cre/+^* mutants and we observe a later phenotype, mostly affecting the IL. As found in the AL, the proportion of differentiated cells is reduced, but strikingly there is a complete downregulation of the melanotroph fate pioneer factor PAX7. This results in a switch of identity of the IL POMC-positive cells to ectopic corticotrophs. When we rescue the proliferation defect in *Sox2* mutants by removing *p27*, we do not observe a rescue of IL melanotroph identity. These results demonstrate that SOX2 has two independent functions during pituitary morphogenesis, proliferation of progenitors and later cell fate acquisition in the IL.

We have examined the expression of key transcription factors required in RP progenitors, and are co-expressed with SOX2, to better understand its early role. Despite a severe hypoplastic phenotype in *Sox2^fl/fl^;Foxg1^Cre/+^* mutants, the early pattern and expression levels of *Pitx2*, and LHX3 protein and gene are not affected by the loss of SOX2. In contrast, we observe a clear downregulation of *Prop1*, as reported previously (Jayakody et al., 2012), and of *Hesx1*, which was suspected of being a SOX2 target (Kelberman et al., 2006). Jayakody et al. used *Hesx1^Cre^* to delete *Sox2*, which could have given misleading results given that (i) the *Cre* driver depends on the product of its intended target gene (*Sox2*) for its own expression and (ii) *Hesx1^Cre^;Sox2^fl/fl^* embryos are already lacking one functional allele of *Hesx1*. While their results and this study both obtain a similar hypoplasia in the anterior lobe, Jayakody et al. did not report any IL phenotype. HESX1 and PROP1 are sequentially expressed in RP progenitors, with a slight overlap. In contrast with *Sox2* mutant phenotypes, deletion of *Hesx1* is mostly associated with hyperproliferation in RP (Dasen et al., 2001) whereas *Prop1* loss results in accumulation of progenitors near the cleft because these are unable to differentiate and populate the developing AL. PROP1, the first exclusive marker of pituitary identity, therefore promotes progenitor commitment (Ward et al., 2005). The significance of a direct or indirect regulatory role for SOX2 in HESX1 and PROP1 expression is unclear, but it might reflect an involvement of SOX2 in establishment and/or maintenance of pituitary identity. SOX2 and PROP1 remain co-expressed in rat pituitary progenitors/SC until PROP1 expression fades in adults (Yoshida et al., 2009, 2011), and it would be of interest to determine whether SOX2 is continuously required to maintain normal levels of PROP1 expression.

In contrast, the significance of *Six6* regulation by SOX2 is very clear, but the molecular mechanisms appear complex. SOX2 directly regulates *Six6* in the VD (Lee et al., 2012), but the enhancer bound by SOX2 in the VD is not active in RP (Lee et al., 2012). The complexity of the regulatory network is further revealed by the maintenance of SIX6 expression after *Sox2* deletion in IL melanotrophs using *Pomc-Cre* (Langlais et al., 2013) and in late progenitors/SCs, using *Sox9^CreERT2^* (Rizzoti et al., 2013). We expect the properties of progenitors to be affected by the loss of SOX2. Therefore, the maintenance of SIX6 expression after *Sox2* deletion in progenitors might rather be linked to their progression toward commitment and/or differentiation once SOX2 is downregulated, rather than simply reflect SOX2-independent regulation of SIX6 in late versus early progenitors. The specific roles of SOX2 in the variety of cell types where it is expressed rely on its interaction with different partners, and the nature of the complexes that bind DNA. Therefore these results suggest that SOX2 has a specific partner in RP progenitors for *Six6* transactivation, but that SOX2 then becomes redundant in differentiated and differentiating cells.

**Fig. 7.**
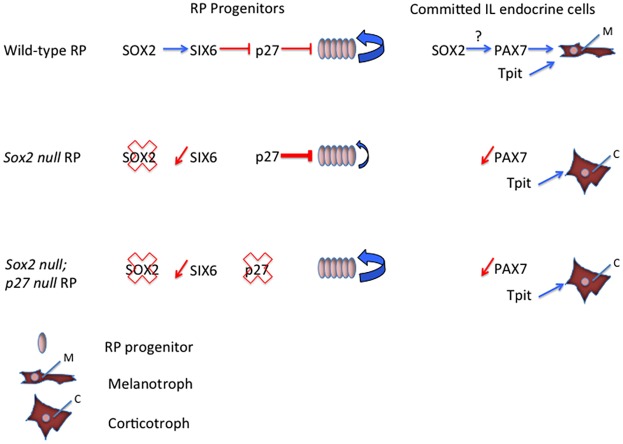
**Model representing the roles of SOX2 during pituitary morphogenesis.** In RP progenitors, SOX2 is likely to directly activate *Six6* expression. SIX6 has been shown to repress P27 expression, hence promoting progenitor proliferation (Li et al., 2002). In the absence of SOX2, we show that SIX6 is downregulated, whereas P27 appears to be upregulated (data not shown; Jayakody et al., 2012); as a result, cell proliferation is reduced. In support of this hypothesis, we show that ablating *p27* in *Sox2* mutants results in a rescue of proliferation. In melanotroph precursors, we show that SOX2 is required for PAX7 upregulation, but not TPIT expression as *Sox2* mutants develop ectopic TPIT^+^; POMC^+^ corticotrophs in IL. This is independent of the role of SOX2 in cell proliferation as ablating P27 has no effect on the emergence of ectopic IL corticotrophs in *Sox2* mutants.

SIX6 associates with DACH corepressors to downregulate transcription of *p27* in the eye, and hence indirectly promote proliferation (Li et al., 2002). In RP, SIX6 has been proposed to positively regulate progenitor proliferation by the same mechanism (Li et al., 2002). This fits well with the observed upregulation of P27 in *Sox2* mutants (data not shown; Jayakody et al., 2012), because SIX6 is essentially absent. Therefore, SOX2 might promote RP progenitor proliferation indirectly, through upregulation of SIX6, which in turn represses P27 (Fig. 7). SIX6 is probably not the only relevant target of SOX2 in RP progenitors, but we expect it to play a significant role because *Six6* (Li et al., 2002) and *Sox2* mutant RP phenotypes are relatively similar. Once P27 is upregulated in MSH-secreting cells, we hypothesize that it can recruit co-repressors to downregulate expression of *Sox2*, as shown *in vitro* (Li et al., 2012). However, low levels of SOX2 persist in melanotrophs, and elevated levels of SOX2 in these and/or SC, as observed in *p27*-null mice, result in IL tumor development (Li et al., 2012). It is important to determine which of these two cell types is most sensitive to alteration in SOX2 expression to understand mechanisms of tumor formation.

In an attempt to restore proliferation in *Sox2* mutants, we decided to delete *p27*. In agreement with our hypothesis we observe a clear rescue of proliferation in double mutants. However, the differentiated cells in the IL of *Sox2;p27* double mutants are still ectopic corticotrophs. This is a strong argument in favor of a second role for SOX2 in acquisition of melanotroph identity. Our results further suggest that SOX2 is required for upregulation, but not maintenance, of the melanotroph pioneer transcription factor PAX7. The molecular mechanisms underlying this requirement are unknown. We analyzed 143 kb surrounding *Pax7* for evolutionary conserved regulatory elements and could not find any SOX2 consensus binding sites within these. Moreover, ChIP-seq experiments in an altered AtT20 corticotroph cell line, engineered to overexpress SOX2, did not reveal any binding of SOX2 at the *Pax7* locus (J. Drouin, personal communication). Further investigations are required to determine whether SOX2 requires a specific partner to upregulate expression of *Pax7*, perhaps via variant binding sites, or alternatively to demonstrate that regulation is indirect.

In conclusion, our work has uncovered a new role for SOX2 in melanotroph cell fate acquisition, independently of its early role in promoting progenitor proliferation (Fig. 7). We also reveal that SOX2 is maintained at low levels in melanotrophs where its expression is likely regulated by P27 (Li et al., 2012). It is important to understand the significance of this, because it might be relevant for development of tumors in the IL. Finally, we have better placed SOX2 in the hierarchy of transcription factors involved in early development of the pituitary. This might aid clinical diagnosis of pituitary defects and be relevant for regenerative medicine approaches for their treatment (Suga et al., 2011; Dincer et al., 2013).

## MATERIALS AND METHODS

### Ethics statement

All experiments carried out on mice were approved under the UK Animal (scientific procedures) Act (Project licenses 80/2405 and 70/8560).

### Mice

*Sox2^fl/fl^* (Taranova et al., 2006), *p27^−/−^* (Fero et al., 1996), *Nkx3.1^Cre/+^* (Y.P.H., S. M. Price, Z. Chen, W. A. Banach-Petrosky, C. Abate-Shen and M.M.S., unpublished), *Pomc-Cre* (Langlais et al., 2013) and *Sox9^ires−CreERT2/+^* (Furuyama et al., 2010) were maintained on mixed backgrounds. *Foxg1^Cre/+^;Sox2^fl/fl^* (Hebert and McConnell, 2000; Taranova et al., 2006) animals were maintained on 129S8 background and *R26R^eYFP/eYFP^* (Srinivas et al., 2001) on C57Bl6 background. To generate *Sox2* mutants, *Sox2^fl/+^; Cre*/+ animals were generated and subsequently bred with *Sox2^fl/+^* or *Sox2^fl/fl^* animals. Cre activity in *Sox9^ires-CreERT2/+^;Sox2^fl/fl^* embryos was induced by tamoxifen treatment (0.2 mg/g/day) in pregnant females at 13.5 dpc.

### Immunohistochemistry and *in situ* hybridization

BrdU and EdU were injected into the peritoneum of pregnant mice at a concentration of, respectively, 100 µg and 30 µg/g body weight. Following a 1 h pulse, embryos were harvested. Generally, embryos were harvested and fixed by immersion in 4% PFA at 4°C, a few hours for immunofluorescence and overnight for *in situ* hybridization. Immunofluorescence was performed on at least three embryos for each genotype, on cryosections as described (Rizzoti et al., 2004), for antibodies see Table S1. EdU assays were performed using a kit (Invitrogen) following manufacturer's instructions. *In situ* hybridizations were performed as described (Rizzoti et al., 2004) using *Pitx2* (Meyers and Martin, 1999) and *Prop1* (Sajedi et al., 2008) probes.

### RNA extraction and real-time quantitative PCR

RNA was extracted from embryonic heads using Trizol (Ambion). A reverse transcription using Superscript II (Invitrogen) was performed on 5 µg samples after DNase digestion. Real-time quantitative PCR was performed using Platinum SYBR Green (Invitrogen) and RT^2^ qPCR Primer assays for mouse Hesx1 and Lhx3 (Qiagen) on a 7500 Real-Time PCR System (AB Applied Biosystems). Expression levels were normalized to those of *GAPDH*. Data was analyzed using the ΔΔCt method (Livak and Schmittgen, 2001).

### Statistical analysis

BrdU- or EdU-positive nuclei and POMC-positive cells were quantified as a percentage of DAPI-positive nuclei, counted in at least three embryos/experiment on three different sections/embryo. Quantification was restricted to the dorsal region of RP where *Nkx3.1^Cre^* is active in *Sox2^fl/fl^;Nkx3.1^Cre/+^* embryos. BrdU was counted throughout RP in *Sox2^fl/fl^;Foxg1^Cre/+^* embryos as *Foxg1^Cre^* activity is ubiquitous in RP. The IL was defined by morphology. Student’s *t*-tests were performed for statistical analysis using Prism software (GraphPad), and means±standard deviations (s.d.) are presented. Angular transformations were applied to compare percentages.
